# DGC-specific *RHOA* mutations maintained cancer cell survival and promoted cell migration via ROCK inactivation

**DOI:** 10.18632/oncotarget.25269

**Published:** 2018-05-01

**Authors:** Takashi Nishizawa, Kiyotaka Nakano, Aya Harada, Miwako Kakiuchi, Shin-Ichi Funahashi, Masami Suzuki, Shumpei Ishikawa, Hiroyuki Aburatani

**Affiliations:** ^1^ Department for Research, Forerunner Pharma Research Co., Ltd., Tokyo, Japan; ^2^ Genome Science Division, Research Center for Advanced Science and Technology, The University of Tokyo, Tokyo, Japan; ^3^ Department of Genomic Pathology, Medical Research Institute, Tokyo Medical and Dental University, Tokyo, Japan

**Keywords:** RHOA, ROCK, diffuse gastric cancer, mutation, ARHGAP

## Abstract

*RHOA* missense mutations exist specifically in diffuse type gastric cancers (DGC) and are considered one of the DGC driver genes, but it is not fully understood how *RHOA* mutations contribute to DGC development. Here we examined how *RHOA* mutations affect cancer cell survival and cell motility. We revealed that cell survival was maintained by specific mutation sites, namely G17, Y42, and L57. Because these functional mutations suppressed MLC2 phosphorylation and actin stress fiber formation, we realized they act in a dominant-negative fashion against the ROCK pathway. Through the same inactivating mechanism that maintained cell survival, *RHOA* mutations also increased cell migration activity. Cell survival and migration studies on *CLDN18-ARHGAP* (*CLG*) fusions, which are known to be mutually exclusive to *RHOA* mutations, showed that *CLG* fusions complemented cell survival under *RHOA* knockdown condition and also induced cell migration. Site-directed mutagenesis analysis revealed the importance of the GAP domain and indicated that *CLG* fusions maintained RHOA in the inactive form. Taken together, these findings show that the inactivation of ROCK would be a key step in DGC development, so ROCK activation might provide novel therapeutic opportunities.

## INTRODUCTION

Diffuse-type gastric cancers (DGC) account for approximately 30% of all gastric cancers and are characterized by poorly differentiated adenocarcinoma with a worse prognosis than the intestinal type [1–3]. DGC infiltrate into adjacent stromal tissues, spread without clear polyps or ulcers, and frequently show intraperitoneal metastasis [4, 5]. Comprehensive genomic sequencing studies to identify DGC-specific genetic alterations, including our previous study, have shown that 14–25% DGC patients carry *RHOA* missense mutations, such as R5W, G17E, Y42C, and L57V [6–8].

RHOA is a small GTPase that belongs to the RHO family and has various biological functions, such as cytokinesis, cell motility, and tissue development [9–11]. RHOA cycles between the GDP-bound inactive form and the GTP-bound active form under the control of regulatory proteins like guanine nucleotide exchange factors (GEFs) and GTPase-activating proteins (GAPs). These regulatory proteins induce conformational change in RHOA to allow binding to substrates named effector proteins, one of which is Rho-associated protein kinase (ROCK). ROCK-LIMK-CFL1 signaling contributes to actin filament stabilization, while ROCK-MLCP-MLC signaling promotes actomyosin formation [12, 13].

In our previous work, we observed that a knockdown of *RHOA* in *RHOA*-mutated cancer cell lines represses cell survival significantly [6]. Wang et al. also reported that introducing *RHOA* mutations, Y42 and L57V, to a murine intestinal organoid promotes cell survival [7]. Moreover, a comprehensive investigation of TCGA revealed that negative regulators of RHOA, *GAP6* and *GAP26*, fused with the tight junction membrane protein *CLDN18* in a DGC-specific manner [8]. The frequency of *CLDN18-GAP* (*CLG*) fusions is 15% in DGC and, interestingly, *RHOA* mutations and *CLG* fusions are mutually exclusive. Although these results suggest that a dysregulated RHOA signal is related to DGC development, the details remain to be understood. In this study, we explored the contribution of *RHOA* mutations to DGC development, focusing on cell survival and also on cell motility, which is one of the features of DGC. Furthermore, we evaluated the functional relationship between *RHOA* mutations and *CLG* fusions.

## RESULTS

### *RHOA*-siRNA treatment inhibited 3D cell survival of *RHOA*-mutated cell lines in a mutation-dependent manner

**Figure 1:**
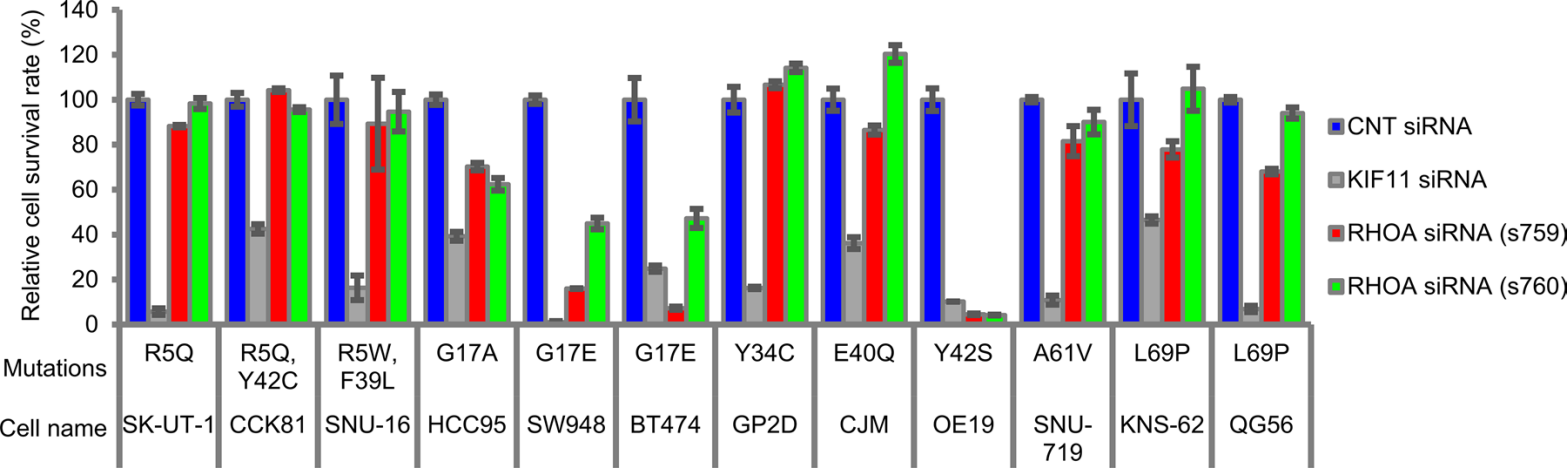
Cell survival rate of various types of cancer cell lines treated with *RHOA*-siRNAs Endogenous *RHOA*-mutated cancer cell lines were seeded in a low attachment plate and then treated with each siRNA for 7 days. The viable cells were measured by CellTiter-Glo 3D Cell Viability Assay. Data are shown as mean ± SD (*n* = 3). Cell selection criteria (see Materials & Methods) ensured the knockdown efficiency of siRNAs.

To identify which mutation sites contribute to cancer cell survival, we selected *RHOA*-mutated cell lines from public databases (Supplementary Table 1) and chose 12 cell lines. In 3D culture conditions, we evaluated the inhibition efficacy of *RHOA*-siRNAs on cell survival and observed significant *RHOA*-siRNA-dependent inhibition in cell lines HCC95, SW948, BT474, and OE19, which carried G17 or Y42 single mutations (Figure 1). On the other hand, cell survival inhibition seen in R5, Y34, E40, A61, and A69 single mutants or in R5/Y42 or R5/F39 double mutants was less clear, and cell survival of 4 *RHOA*-WT cell lines was not inhibited (Supplementary Figure 1).

### Mutated *RHOA* contributed cell survival, and G17V, Y42C, Y42S, and L57V mutations showed functional complementarity to G17E

**Figure 2:**
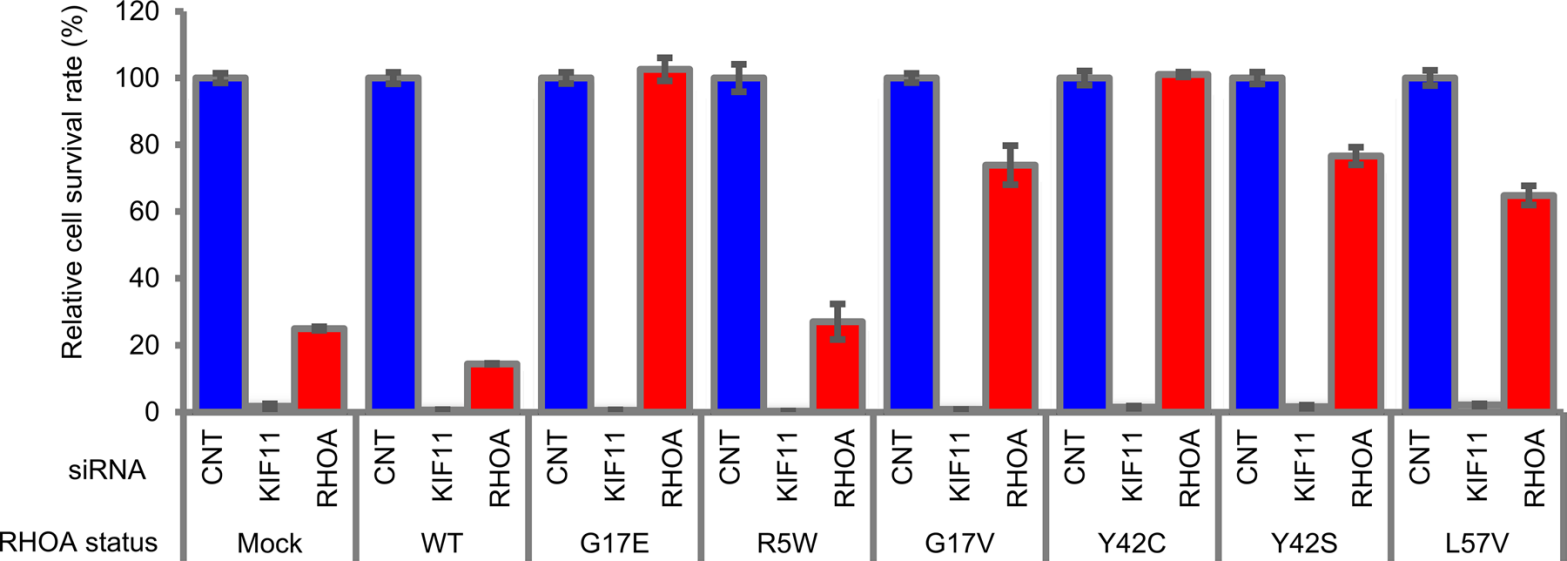
Rescue study of *RHOA*-siRNA-dependent inhibition of cell survival in SW948 SW948 was transfected with WT and each mutated *RHOA*. Cell survival rate of obtained transfectants was evaluated as described in Figure 1. siRNA ID: s759 was used for *RHOA*-siRNA. Data are shown as mean ± SD (*n* = 3). Protein expression levels are shown in Supplementary Figure 8A.

Next, we investigated which types of *RHOA* contribute to cell survival in SW948 cells, which express G17E- and WT-*RHOA* heterogeneously. We used stable SW948 transfectants that expressed siRNA-resistant G17E- and WT-*RHOA*, and then evaluated whether *RHOA*-siRNA continued to inhibit cell survival or not (Figure 2). While the introduced G17E mutation restored cell survival, the WT did not.

We also checked the functional complementarity with mutations that were found in clinical specimens. Because L57V-mutated cancer cell lines were unavailable commercially, we added the mutation for this experiment. The siRNA-dependent inhibition of cell survival was cancelled not only by the introduction of G17E, but also of G17V, Y42C, Y42S, and L57V; however, it was not cancelled by the R5W mutant (Figure 2). To confirm these results, we also expressed abundant mutated *RHOA* transiently in SW948 to evaluate cell survival, and the same tendency was observed (Supplementary Figure 2B). These results revealed that the mutations in G17, Y42, and L57 also contributed to cancer cell survival.

### *RHOA*-knockdown in *RHOA*-mutated SW948 induced ROCK activation via RHOB

**Figure 3:**
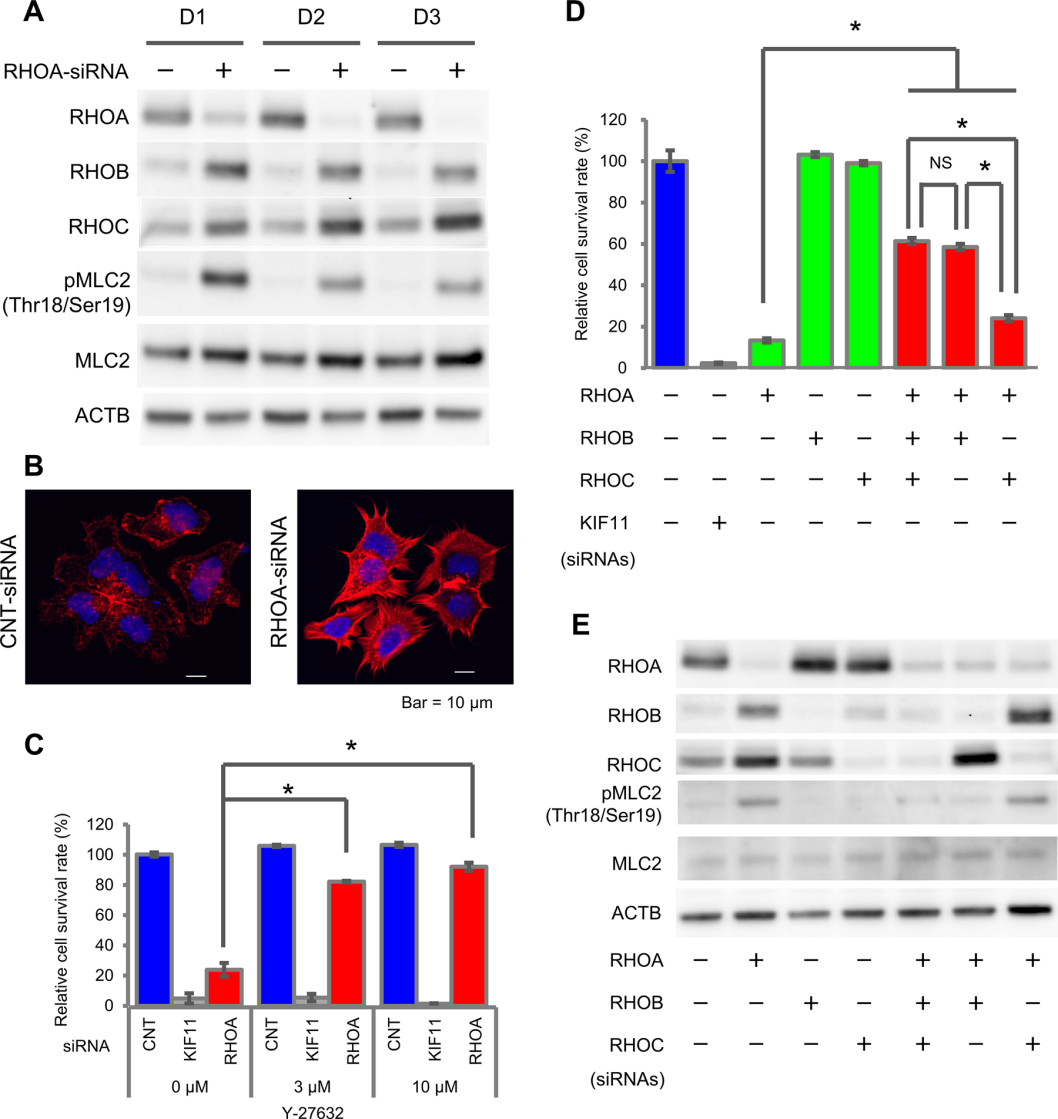
Activation of ROCK signaling by RHOA knockdown in SW948 (**A**) Expression of RHOA, RHOB, and RHOC, and phosphorylation of MLC2 in SW948 treated with 1 nM of *RHOA*-siRNA. Proteins were harvested on days 1, 2, and 3 after siRNA treatment. The protein expression levels were detected using western blotting. (**B**) Actin stress fibers of SW948 treated with 1 nM of *RHOA*-siRNA. Actin stress fibers were stained with Rhodamine Phalloidin, and DAPI was used for nuclear staining. Stained cells were analyzed with confocal fluorescence microscopy. Representative images of three independent chambers are shown. Details of immunocytochemistry are described in Materials and Methods. Scale bar shows 10 μm. (**C**) Restoration of cell survival by a ROCK inhibitor, Y-27632. SW948 was cultivated for 7 days with 3 μM or 10 μM of Y-27632. The relative cell survival rate is shown as a percentage of that in the control-siRNA-treated SW948 that was not treated with Y-27632. Data are shown as mean ± SD (*n* = 3). Significance compared with the Y-27632 non-treated group between *RHOA*-siRNA-treated groups was determined by Student’s *t*-test. ^*^p < 0.05. (**D**) Restoration of cell survival by *RHOB/RHOC*-siRNAs. SW948 was cultivated for 7 days with *RHOA*-siRNA (1 μM) and/or *RHOB*-siRNA (1 μM) and/or *RHOC*-siRNA (0.2 μM). Cell survival rate of obtained transfectants was evaluated as described in Figure 1. Data are shown as mean ± SD (*n* = 3). Significant differences between *RHO*-siRNA-treated groups were determined by Student’s *t*-test. ^*^p < 0.05. (**E**) Protein expression of cells tested in (D).

To reveal the signal cascade that contributes to cell survival, we analyzed the time course of the ROCK pathway, which is one of the major RHOA signaling pathways, after *RHOA*-siRNA treatment. We evaluated the change in other RHO family proteins, RHOB and RHOC, and in signal molecules, ROCK1/2, MLC2, MYPT1, LIMK1/2, and CFL1. RHOA protein expression was knocked down significantly on Day 1 after *RHOA*-siRNA treatment and was almost completely depleted on Day 2 (Figure 3A), while the expression of RHOB and RHOC proteins was accordingly elevated. *RHOA*-siRNA treatment elevated the phosphorylation of MLC2 (Thr18/Ser19). We also noted that MYPT1 (Thr696 and Thr853), which is a phosphatase of MLC2, was not phosphorylated (Supplementary Figure 3), and LIMK1 (Thr508)/LIMK2 (Thr505) and CFL1 (Ser3) were constantly phosphorylated independently of *RHOA*-siRNA. From these results, we assumed that RHOA depletion induced ROCK-MLC2 signal activation. To clarify whether the ROCK activation induced by RHOA depletion affected the cytoskeleton or not, we stained for actin stress fiber. After *RHOA*-siRNA treatment, the formation of actin stress fiber was clearly increased and the shape was spiky (Figure 3B). This result verified that a knockdown of *RHOA* activated ROCK and stimulated actin stress fiber formation. Next, to investigate whether the suppression of ROCK would promote cell survival or not, we evaluated the effect of a ROCK1/2 inhibitor, Y-27632, on the cell survival of SW948. After treatment with Y-27632, the survival rate of *RHOA*-siRNA-treated cells recovered significantly from 24% (non-treatment) to 82% (3 μM) and 92% (10 μM) (Figure 3C). Y-27632 also inhibited the phosphorylation of MLC2 (Supplementary Figure 4). We revealed that inactivation of ROCK promoted cell survival. Overall, this series of results revealed that *RHOA* mutations keep suppressing ROCK activation, so their effect on ROCK is dominant-negative.

We hypothesized that ROCK reactivation would be induced by RHOB and/or RHOC, because the expression of these RHO molecules was elevated after *RHOA*-siRNA treatment. To evaluate this hypothesis, we used *RHOB* and/or *RHOC*-siRNAs for a rescue study. The survival rate of *RHOA*-siRNA-treated cells increased significantly from 13% to 61% (+*RHOB-* and *RHOC*-siRNAs), 59% (+*RHOB*-siRNA) and 24% (+*RHOC*-siRNA) (Figure 3D). *RHOB*-siRNA inhibited the phosphorylation of MLC2 induced by *RHOA*-siRNA treatment (Figure 3E). These results revealed that ROCK activation was induced by RHOB in SW948.

### The inhibition of cell survival by *RHOA*-siRNA was cancelled by *CLG* fusions and *GAPs*, but not by *CLDN18*

**Figure 4:**
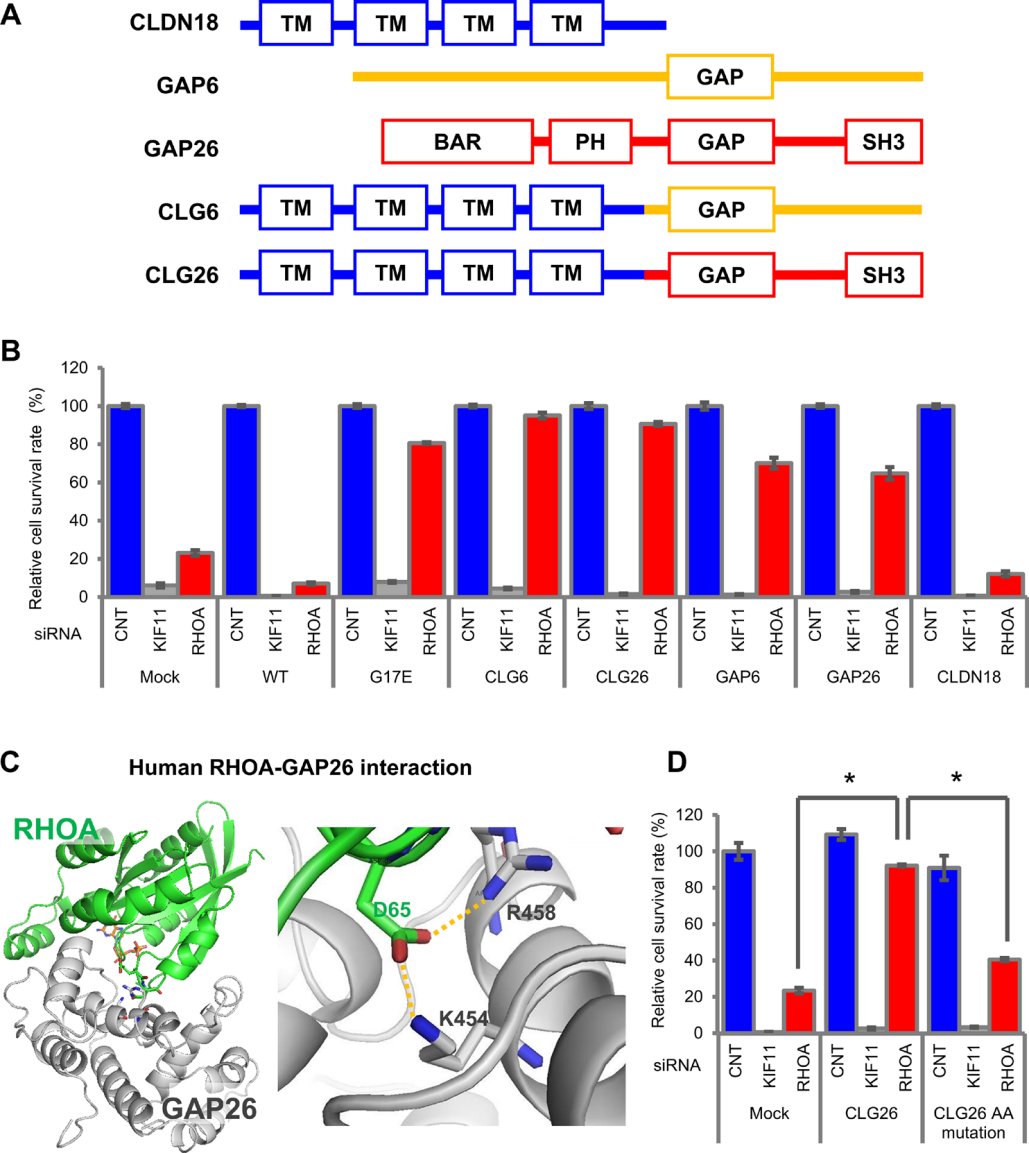
Cell survival promoted by *CLG* fusions in SW948 (**A**) Domain structure of CLG fusions. (**B**) Restoration of cell survival by *CLG* fusions. Cell survival rate was evaluated as described in Figure 3C. (**C**) Structure of RHOA and GAP complex inferred from homology modeling of PDB: 1TX4. RHOA in green and GAP26 in silver are shown in stick form. Hydrogen bonds are shown by an orange dotted line. A close-up (right) of hydrogen bonds in the overall model (left) is shown. (**D**) Reduction in cell survival activity by GAP domain AA mutations. SW948 was transfected with K454A/R458E double-mutated *CLG26*. Cell survival rate of each obtained transfectant was evaluated as described above. The relative cell survival rate is shown as a percentage of that in the mock transfectants treated with control-siRNA. Data are shown as mean ± SD (*n* = 3). Statistical significance of the CLG26 group compared with *RHOA*-siRNA-treated groups was determined by Student’s *t*-test. ^*^p < 0.05.

To reveal the functional relationship between *RHOA* mutations and *CLG* fusions, we treated stable SW948 transfectants that expressed *CLG* fusions with *RHOA*-siRNA and evaluated the effect on cell survival. The domain structures of CLDN18, GAP6, GAP26, CLG6, and CLG26 are shown in Figure 4A. *RHOA*-siRNA-dependent cell survival inhibition was canceled by *CLG6, CLG26, GAP6*, and *GAP26*, but not by *CLDN18* (Figure 4B). These results indicated that *CLG* fusions complemented *RHOA* mutations, in terms of their effect on cell survival.

Further investigation served to confirm whether survival in cells with *CLG* fusions was dependent on *GAP*, which inactivates *RHOA*. A published report indicated the intensity of the RHOA and GAP26 interaction by showing that mutations of K454 and R458 in *GAP26* remarkably decreased the thermodynamic and kinetic scores [14]. Using 3D modeling, we confirmed that K454 and R458 are important for the interaction between GAP26 and RHOA (Figure 4C) because they form hydrogen bonds to D65 in RHOA. Therefore, we introduced K454A/R458E double-mutated *CLG26* into SW948 and established a stable transfectant in which GAP activity was eliminated. This double mutation in the GAP domain significantly decreased the contribution of *CLG26* to survival (Figure 4D). These results suggested that GAP activity was necessary for cell survival.

### *RHOA* mutations and *CLG* fusions induced migration activity by inactivating ROCK

**Figure 5:**
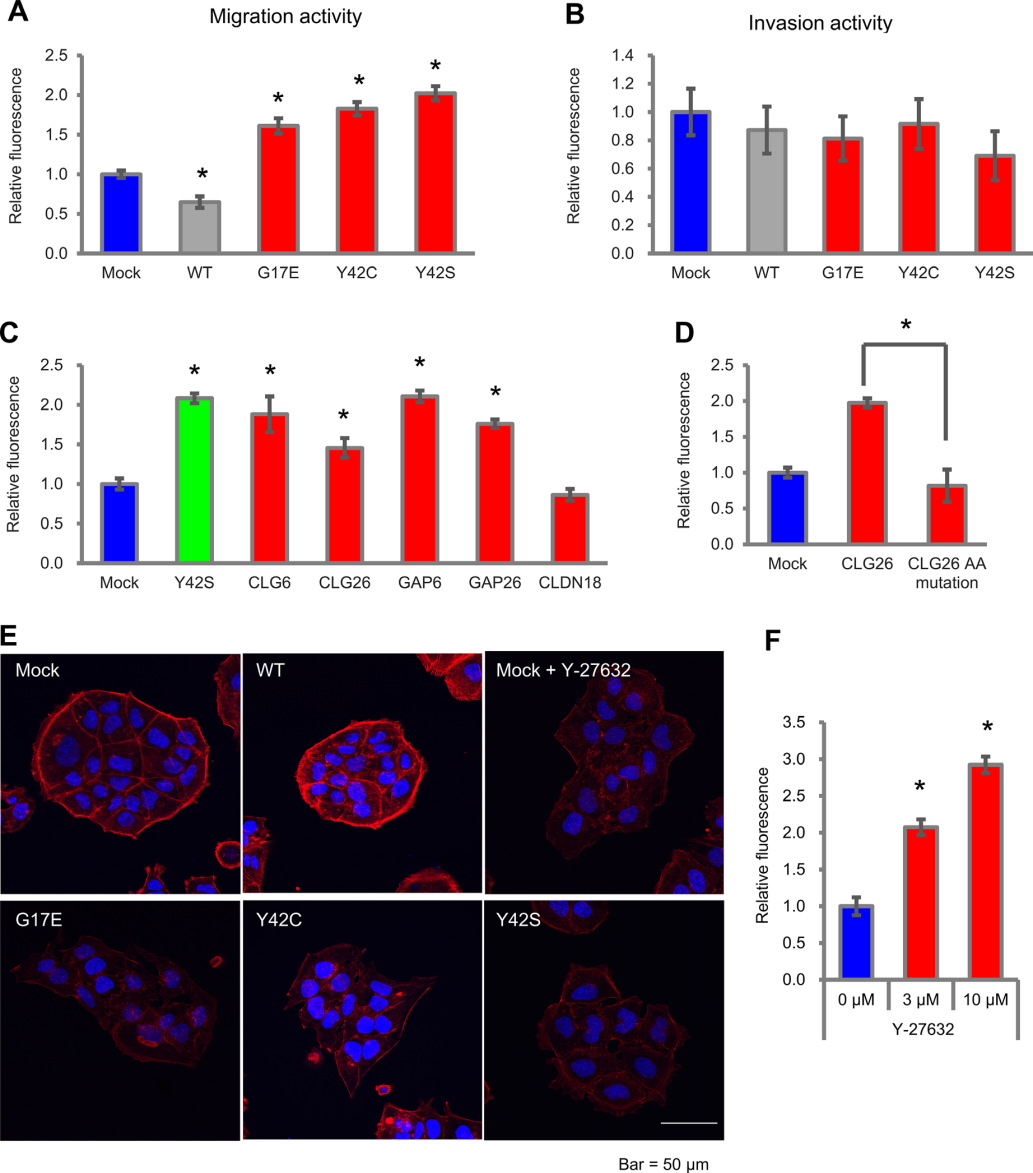
Cell motility in MKN74 cells with *RHOA* mutations and *CLG* fusions (**A**) Cell motility in an uncoated chamber and (**B**) in a Matrigel-coated chamber was measured 48 hrs after plating with MKN74 transfectants of *RHOA* mutations. The migrating cells were stained with calcein AM, and the fluorescence was measured by a plate reader. Data are shown as mean ± SD (*n* = 3). Statistical significance compared with the mock group was determined by Student’s *t*-test. ^*^p < 0.05. Methods of calculating invasion activity are described in Materials and Methods. (**C**) Migration activity of MKN74 transfectants with *CLG* fusions, *GAPs*, and *CLDN18*. Cells were seeded in an uncoated chamber and migrated cells were stained with calcein AM. Data are shown as mean ± SD (*n* = 3). Statistical significance compared with the mock group was determined by Student’s *t*-test. ^*^p < 0.05. (**D**) Reduction of cell migration by GAP domain AA mutations. MKN74 was transfected with K454A/R458E double-mutated *CLG26*. Migration activity was evaluated as described above. (**E**) Actin stress fibers in MKN74 transfectants with *RHOA* mutations. Mock cells were treated with 10 μM of Y-27632. Actin stress fibers were stained with Rhodamine Phalloidin, and DAPI was used for nuclear staining. Stained cells were analyzed with confocal fluorescence microscopy. Representative images from three independent fields of view are shown. Scale bar shows 50 μm. (**F**) Migration activity promoted by a ROCK inhibitor in MKN74 transfectants. Cells were seeded in an uncoated chamber and cultivated for 48 hrs with 3 μM or 10 μM of Y-27632. Migration activity was measured as described above. Data are shown as mean ± SD (*n* = 3). Statistical significance compared with the non-treated group was determined by Student›s *t*-test. ^*^p < 0.05.

Next, we evaluated the effect of *RHOA* mutations on cell motility, which is a feature of DGC. We introduced WT, G17E, Y42C, and Y42S into MKN74, and used the transfectants for migration and invasion assays in a Boyden Chamber. Compared with mock, G17E, Y42C, and Y42S promoted cell migration activity 1.6- to 2.0-fold, whereas WT decreased the migration activity 0.65-fold (Figure 5A). Representative images of the migration assay are shown in Supplementary Figure 5. We also evaluated the invasion activity of these transfectants with a Matrigel-coated chamber, but a clear difference was not observed (Figure 5B).

When we evaluated cell motility in *CLG* fusions, migration activity compared with mock was significantly increased by *CLG6*, *CLG26*, *GAP6*, and *GAP26*, but not by *CLDN18* (Figure 5C). However, when the K454A/R458E double mutation in the GAP domain was introduced to the *CLG26* transfectant, the migration activity was diminished (Figure 5D). We revealed that, similarly to *RHOA* mutations, *CLG* fusions contributed to migration activity in addition to cell survival, and that this contribution was dependent on the GAP activity.

As in the cell survival assays, we clarified the relationship between cell migration and ROCK activation by staining for actin stress fiber to reveal the cytoskeleton of MKN74 transfectants (Figure 5E). The mock transfectant showed clear stress fiber formation localized around cell clusters; on the other hand, G17E, Y42C, and Y42S showed weaker actin stress fiber formation, and their localization around cell clusters was unclear. These cytoskeletal changes induced by *RHOA* mutations were similar to those found in Y-27632-treated cells, which indicates the possibility that the inactivation of ROCK also contributed to migration activity. To verify this hypothesis, we evaluated the migration activity of MKN74 after Y-27632 treatment. As a result, Y-27632 increased migration activity 2.1-fold (3 μM) and 2.9-fold (10 μM) compared with non-treatment (Figure 5F). These results revealed that the inactivation of ROCK promoted cell migration activity.

## DISCUSSION

In this study, we revealed that *RHOA* mutations promoted cancer cell survival and migration activity by inactivating ROCK. At first, the presence of several hotspot amino acids led us to assume that *RHOA* mutations would be gain-of-function mutations, similar to mutations in *RAS* and *RAC* [15–18]. However, contrary to our expectations, the knockdown of *RHOA* induced ROCK activation, and a ROCK inhibitor achieved cell survival similar to that seen in *RHOA* mutations; therefore, we concluded that functional *RHOA* mutations were loss-of-function (LoF) mutations for ROCK activation. Wang et al. reported that the amount of GTP form of RHOA in Y42C and L57V was less than that in WT and G14V in a pull-down assay [7], which would support our conclusion. Although *RB1* and *VHL* are well known as tumor suppressor genes that have LoF mutations, they have no clear hotspots [19]. Despite the presence of hotspots, the *RHOA* mutations were LoF type, and cell lines acquired dominant-negative features when site-specific mutations were introduced. Our analysis demonstrated that the hotspot mutations at G17, Y42, and L57 contributed to cell survival, but not those at R5 and L69. On the other hand, in Burkitt’s lymphoma, R5 mutation was reported to be a hotspot and to suppress RHOA-ROCK signaling [20], which suggests that the mechanism by which *RHOA* mutations induce dominant-negative properties might vary depending on the tumor type or cell type. Our next challenge will be to clarify the mechanism by which each *RHOA* mutant inactivates ROCK signaling in DGC.

Our study provided interesting insights about the mechanism of cell death by *RHOA* knockdown. Firstly, *RHOA* knockdown reactivated the ROCK pathway mainly via RHOB. We observed that the protein expression of RHOB and/or RHOC was induced in other cell lines besides SW948 (Supplementary Figure 6), as have other groups [21], which suggests that homeostasis to keep the total amount of RHOs is generally maintained. RHOs have been previously reported to complement each other functionally [22, 23]. Mutated *RHOA* suppresses ROCK activation, but it was interesting to see that upregulated RHOB after *RHOA* knockdown revitalized ROCK signaling through this complementary mechanism. Secondly, ROCK activation induced cell death in *RHOA* mutated cancer cells. In human ES cells and iPS cells, it has been reported that inactivating the ROCK pathway significantly enhances recovery of cells from cryopreserved stocks in cell culture [24]. Upon dissociation, these cells become vulnerable to apoptosis via a phenomenon called apoptotic membrane blebbing. The molecular mechanism that causes apoptotic membrane blebbing would be ROCK signaling activation [25]; that is to say, the phosphorylation of MLC2 by ROCK induces hyperactivation of actomyosin and leads to dissociation-induced apoptosis. As its name suggests, cancer cells of DGC spread from the epithelial layer and diffuse into gastric stromal tissue. Similarly to ES and iPS cells, inactivation of ROCK might protect these vulnerable cancer cells from apoptotic cell death.

The inactivation of ROCK signaling induced by *RHOA* mutations promoted not only cell survival but also cell migration. *RHOA* mutations decreased the accumulation of actin stress fiber around cell clusters and reduced intercellular adhesion, thus loosening the aggregation of cells. These morphological changes might promote cell migration. This possibility is supported by a report that diminished cell-cell interaction by actomyosin was an important step for collective cell migration, the phenomenon by which a group of cells move in concert without completely losing their cell-cell attachment [26]. Several reports that have investigated the relationship between RHOA and cell motility showed that activation of RHOA by overexpression of WT or the constitutive active form (G14V) promoted cell migration [27, 28]. In contrast, our results showed that the introduction of WT and G14V decreased cell migration, and the dominant-negative mutation (T19N) enhanced cell migration (Supplementary Figure 7). To find out how the different patterns of actin stress fiber accumulation affect cell migration, further time-dependent and cell-type-dependent analyses will be necessary.

In this study, we revealed that *CLG* fusions and *RHOA* mutations share a functional relationship; namely, in promoting cancer cell survival and migration. Our mutagenesis experiments showed that the GAP domain was critical for the function of *CLG* fusions. Originally, *GAPs* have a BAR domain, which works as a feedback mechanism to suppress over-activated GAP activity [29], but *CLG* fusions lose their BAR domain. So we assumed that *CLG* fusions promote hydrolysis of GTP-RHOA to GDP-RHOA and thus inactivate ROCK signaling. Since *RHOA* mutations and *CLG* fusions are both DGC-specific genetic alterations and are mutually exclusive, the inactivation of ROCK signaling would be a key step in the development of DGC. A ROCK signaling activator might show broad therapeutic opportunities for ROCK-inactivated DGC patients.

## MATERIALS AND METHODS

### Cell lines

The human cancer cell lines SK-UT-1, SNU-16, SW948, and BT474 were obtained from the American Type Culture Collection (ATCC); HCC95, SNU-719, SNU-484 and SNU-638 from Korean Cell Line Bank (KCLB); GP2D and OE19 from the European Collection of Animal Cell Cultures (ECACC); CCK81, KNS-62, MKN45 and MKN74 from the Japanese Collection of Research Bioresources (JCRB); CJM from Riken; and QG-56 from IBL. Each cell line was cultured using the medium recommended by the suppliers and maintained in a humidified incubator at 37°C with 5% CO_2_, except for SW948 cells, which were cultured without CO_2_.

### Generation of SW948 and MKN74 cell lines expressing *RHOA* mutants or *CLG* fusion genes

For the rescue studies, silencing mutations were introduced into the *RHOA* coding sequence (NCBI RefSeq Sequence: NM_001664.3) so that introduced *RHOA* were resistant to *RHOA*-siRNAs. cDNA of *CLDN18* (NCBI RefSeq Sequence: NM_001002026.2), *GAP6* (NCBI RefSeq Sequence: NM_013423.2), and *GAP26* (NCBI RefSeq Sequence: NM_001135608.1) coding sequences was amplified in mutation-negative cancer cell lines or a cDNA library of normal human tissue (Ambion). cDNA of *CLG26* fusion gene was amplified by RT-PCR from a fusion-positive gastric cancer clinical specimen. The synthesis of *CLG6* fusion genes that combined cDNAs of *CLDN18* and *GAP6* was referred from a published report [8]. *CLDN18* was fused to *GAP6* and *GAP26* that included the GAP domain. These sequences were inserted into the pLVSIN-CMV vector (Takara). Expression plasmids for each *RHOA* mutant and for *CLG26* mutant with GAP domain were generated using site-directed mutagenesis PCR and the In-Fusion HD Cloning system (Clontech). The mixture of expression vector and ViraPower Lentiviral Packaging Mix (Thermo Fisher) was introduced into Lenti-X 293T cells (Takara) using FuGENE HD Transfection Reagent (Promega). After 48 hrs, the culture medium was harvested and virus particles were concentrated with Lenti-X Concentrator (Takara). Prepared lentivirus was transfected into each cell line with hexadimethrine bromide (final 8 ug/mL). Hygromycin was added to establish stable transfectants at a final concentration of 500 μg/mL for SW948 and 25 μg/mL for MKN74. After 48 hrs of *RHOA*-siRNA treatment, protein expression of the siRNA-resistant RHOA was confirmed by Western blot analysis (results are shown for SW948 in Supplementary Figure 8A and for MKN74 in Supplementary Figure 8B), except for G17E/V, which was confirmed by quantitative RT-PCR (qRT-PCR), because protein expression was faint (Supplementary Figure 8C). As for *CLG* fusion genes, expression was validated by qRT-PCR (results are shown for SW948 in Supplementary Figure 9A, 9B and for MKN74 in Supplementary Figure 9C, 9D, 9E).

### Inhibition and rescue assays of cell survival in 3D conditions

An assay to evaluate the inhibition of cell survival in siRNA-treated cells and a function rescue assay were performed as described previously [6]. In brief, cells were seeded in 96-well ultra-low attachment plates (Corning) in triplicate wells. At the same time, mixtures of siRNA and Lipofectamine RNAiMAX reagent (Thermo Fisher) were added to each well as 0.5 or 1 or 5 nM of siRNA solutions. The sequences of siRNAs are listed in Supplementary Table 2A. As a non-targeting negative control siRNA, Silencer Select Negative Control No.1 siRNA (Thermo Fisher) was used. The investigation of *RHOA* mutated cancer cell lines was utilized public database; CCLE; https://portals.broadinstitute.org/ccle/home and COSMIC; http://cancer.sanger.ac.uk/cosmic. The 12 cell lines shown in Figure 1 were selected based on three criteria; namely, knockdown efficiency of *RHOA*-siRNA (over 75%), cell survival inhibition activity by *KIF11*-siRNA (over 70%), and mutation status, which was confirmed in-house. Each cell line had heterogeneous mutated and WT *RHOA*. In *RHOA* double-mutated cells (KOSC-2, CCK-81, and SNU-16), each mutation existed on different alleles. The viable cells were measured 7 days after siRNA transfection using the CellTiter-Glo 3D Cell Viability Assay (Promega). In the rescue assay, cell survival inhibition assays were performed in SW948 cell lines using siRNA-resistant *RHOA* or treatment with Y-27632 or *RHOB/RHOC*-siRNAs. We confirmed Y-27632 up to 33 μM did not affect SW948 cell survival (Supplementary Figure 10A). For transient expression, we inserted each mutated *RHOA* into a pEBMulti-Neo vector (Wako). Each plasmid was transfected into SW948 cells by electroporation with the Nucleofector system (Lonza). Then the procedure described above was followed. Protein expression in the rescue study is shown in Supplementary Figure 2A.

### qRT-PCR

Cells were seeded in 6-well ultra-low attachment plates (Corning). Total RNA was extracted using the RNeasy Mini Kit (Qiagen). To evaluate *RHOA*, RNAs were extracted after 2 days of *RHOA*-siRNA treatment, and to evaluate *CLG* fusion genes, RNAs were extracted after 2 days of cell seeding. qRT-PCR was performed with Power SYBR Green PCR Master Mix (Applied Biosystems), using the primers. The sequences of primers are listed in Supplementary Table 2B. PCR reactions were performed at 48°C for 30 min and 95°C for 10 min, followed by 40 cycles of 95°C for 15 sec and 60°C for 1 min. Values obtained in qRT-PCR were normalized with *RPS18*.

### Western blot analysis

Two days after transfection, cells were also lysed in RIPA buffer (Wako) supplemented with a protease inhibitor cocktail (Roche) and the phosphatase inhibitor PhosSTOP (Roche), and concentrations of the extracts were estimated with a DC protein assay (Bio-Rad). Total cell extract (3–5 μg of protein per lane) was subjected to sodium dodecyl sulfate polyacrylamide gel electrophoresis, and the separated proteins were electrophoretically transferred to Immobilon-P membranes (Millipore). After blocking in Blocking One (Nacalai Tesque), the membranes were incubated in primary antibodies against RHOA, RHOB, RHOC, phospho-CFL1 (Ser3), CFL1, phospho-MLC2 (Thr18/Ser19), MLC2, phospho-LIMK1 (Thr508)/LIMK2 (Thr505), LIMK1, LIMK2, phospho-MYPT1 (Thr696), phospho-MYPT1 (Thr853), MYPT1 (Cell Signaling, #2117, #2098, #3430, #3313, #5175, #3674, #8505, #3841, #3842, #3845, #5163, #4563, #8574), and ACTB (Sigma-Aldrich, A1978). ACTB was used as an internal control.

### Structural analysis

The homology modelling of RHOA-GAP26 was constructed from an X-ray crystallographic structure of the RHOA-GAP1 complex (PDB: 1TX4; https://www.rcsb.org/pdb/home/home.do) using MOE2014 software (Chemical Computing Group). The figure of the RHOA-GAP26 complex was drawn using PyMol v4.2.0 software (Schrödinger).

### Migration and invasion assay

The MKN74 cell line was selected because it originated as differentiated gastric cancer, had *RHOA*-WT with no clear oncogenes (e.g. *KRAS, FGFR2, HER2* etc.), and was easy to handle for transfection. FluoroBlok Multiwell Insert Systems with an 8-μm pore size (Corning) were used to perform the cell migration assay for MKN74 transfectants. The cells were seeded on top of the filter inserts in 1% FBS medium. Then the inserts were placed into the lower chamber, which was loaded with 10% FBS medium. Following incubation for 48 hrs, the cells that traversed the filter were stained with calcein AM (Dojindo), and the fluorescence was read by EnVision Multilabel Reader (PerkinElmer). Stained cells were analyzed with an IX83 Inverted Microscope (Olympus) using a UPLFLN 4X PH objective lens. Photo data processing was performed by Olympus cellSens Dimension software ver 1.15 (Olympus). For invasion, BioCoat Tumor Invasion Multiwell Plates with 8-μm pore size (Corning) were used. Invasion plates were re-hydrated with FBS-free media at 37°C for 2 hrs. After that, the procedure was the same as the migration assay. Invasion activity was calculated according to the maker’s protocol (invasion activity = mean number of invading cells/mean number of migrating cells). We confirmed Y-27632 up to 100 μM did not affect MKN74 cell survival (Supplementary Figure 10B).

### Immunocytochemistry and confocal microscopy

Cells were seeded on Nunc Lab-Tek II CC2 Chamber Slide Systems (Thermo Fisher) and either siRNAs were added simultaneously or Y-27632 was added after 24 hrs incubation. 48 hrs later, cells were fixed with 4% PFA, permeabilized in 0.5% Triton X-100/PBS, and stained with Rhodamine Phalloidin (Thermo Fisher). DAPI was used for nuclear staining. Stained cells were analyzed with the A1 confocal fluorescence microscopy system (Nikon) using a CFI Apochromat Lambda S 60x Oil lens. Photo data processing was performed by NIS-Elements software (Nikon).

## SUPPLEMENTARY MATERIALS FIGURES AND TABLES



## Abbreviations

DGC: diffuse type gastric cancers
GEF: guanine nucleotide exchange factor
GAP: GTPase-activating protein
ROCK: Rho-associated protein kinase
MLC2: Myosin regulatory light chain 2
MYPT1: myosin phosphatase targeting subunit 1
LIMK: LIM domain kinase
CFL1: Cofilin-1
CLDN: Claudin
CLG: CLDN18-ARHGAP
ACTB: Beta-actin
TM: transmembrane
LoF: loss-of-function
RHO-GDI: RHO GDP-dissociation inhibitor
WT: wild type
