# Supplementary material for: DGC-specific RHOA mutations maintained cancer cell survival and promoted cell migration via ROCK inactivation
Source: Oncotarget. 2018 May 1;9(33):23198–207. doi: 10.18632/oncotarget.25269 (PMC5955407; doi:10.18632/oncotarget.25269)
Supplement: Supplementary file 1 [file oncotarget-09-23198-s001.pdf]

DGC-specific *RHOA* mutations maintained cancer cell survival and promoted cell migration via ROCK inactivation

SUPPLEMENTARY MATERIALS

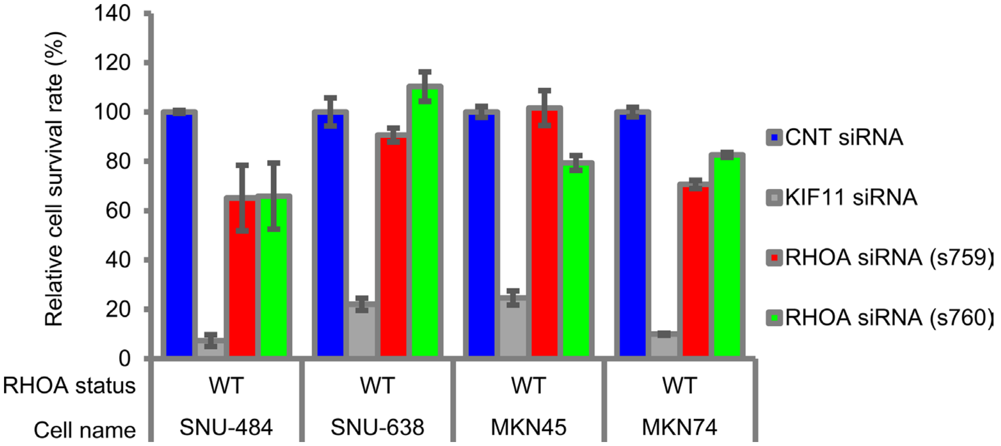

Supplementary Figure 1: Inhibition by *RHOA*-siRNAs of cell survival in *RHOA*-WT gastric cancer cell lines. Cell lines were seeded in a low attachment plate and then treated with each siRNA for 7 days. The viable cells were measured by a CellTiter-Glo 3D Cell Viability Assay. Data are shown as mean ± SD (*n* = 3).

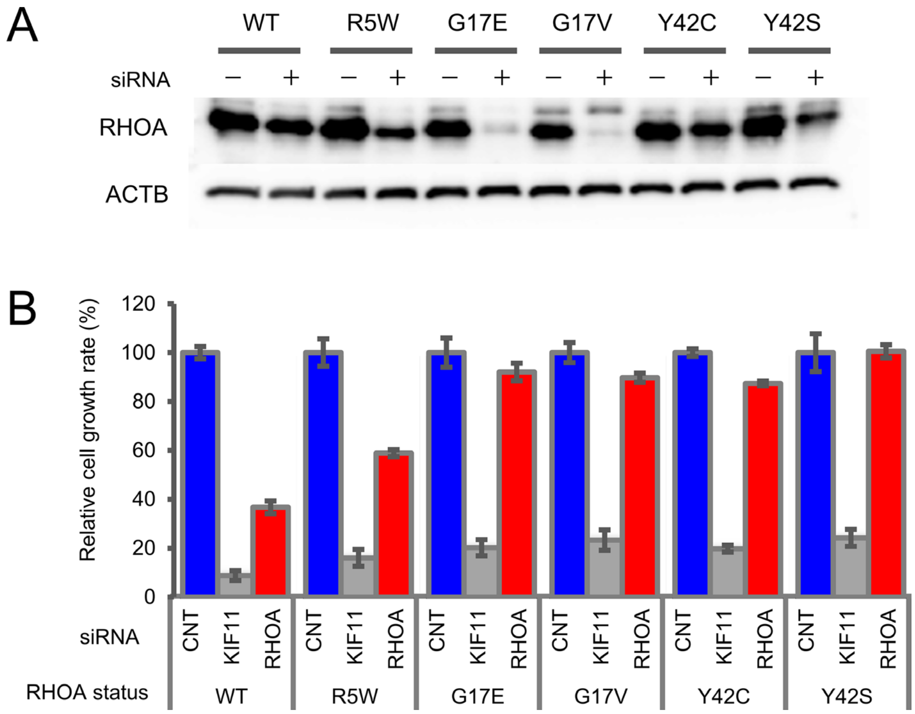

Supplementary Figure 2: Rescue study of *RHOA*-siRNA-dependent inhibition of cell survival in SW948 cells that had been transfected to transiently express abundant WT *RHOA* and each mutated *RHOA*. (**A**) Protein expression of each transfectant. Each vector plasmid was transfected into SW948 cells by electroporation. 2 days after electroporation, cells were seeded in a 6-well plate and then *RHOA*-siRNA was added. After 2 days incubation, proteins were eluted and RHOA protein expression was detected by western blotting. (**B**) Cell survival rate of obtained transfectants was evaluated as described in Figure 1. siRNA ID: s759 was used for *RHOA*-siRNA. Data are shown as mean ± SD (*n* = 3).

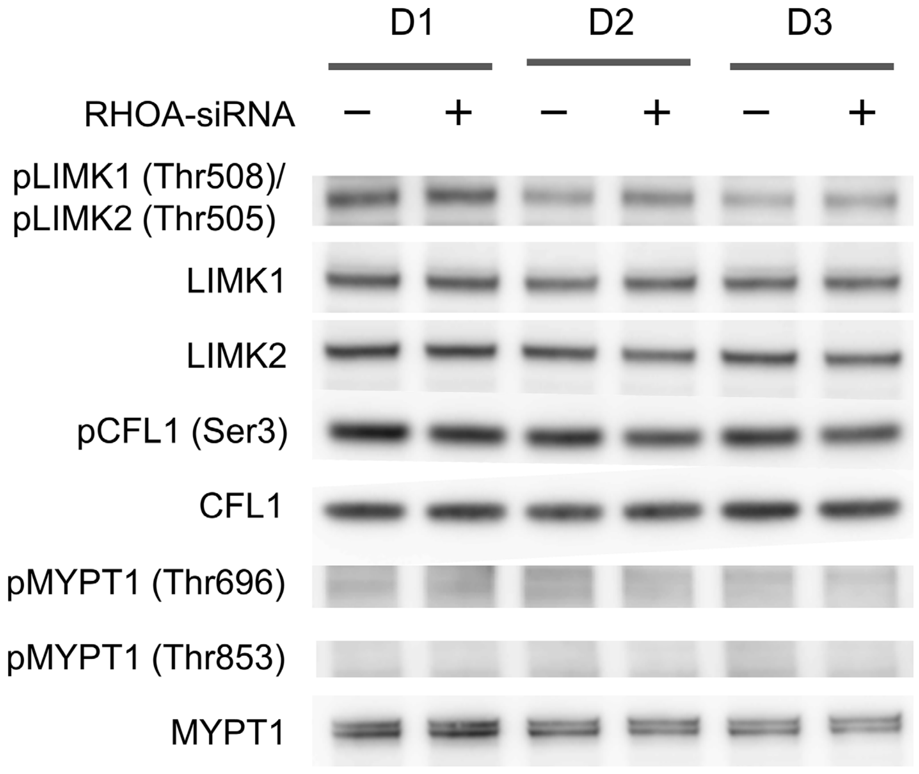

Supplementary Figure 3: Phosphorylation of LIMK1, LIMK2, CFL1, and MYPT1 in SW948 treated with 1 nM of *RHOA*-siRNA. Proteins were harvested on days 1, 2, and 3 after siRNA treatment. The protein expression levels were detected using western blotting.

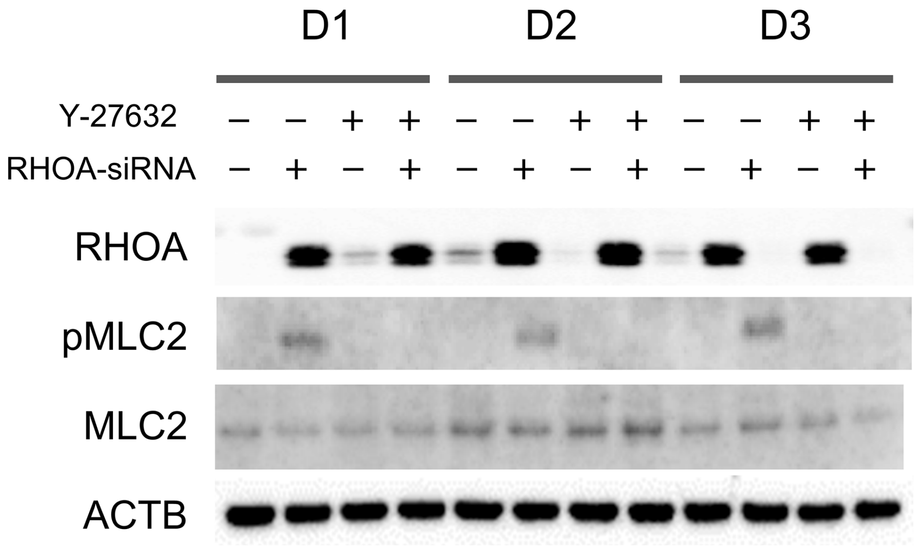

Supplementary Figure 4: Suppression of MLC2 phosphorylation by a ROCK inhibitor. SW948 was seeded in a 6-well plate and then *RHOA*-siRNA or 10 µM of Y-27632 was added. Proteins were harvested on days 1, 2, and 3 after siRNA treatment. The protein expression levels were detected using western blotting.

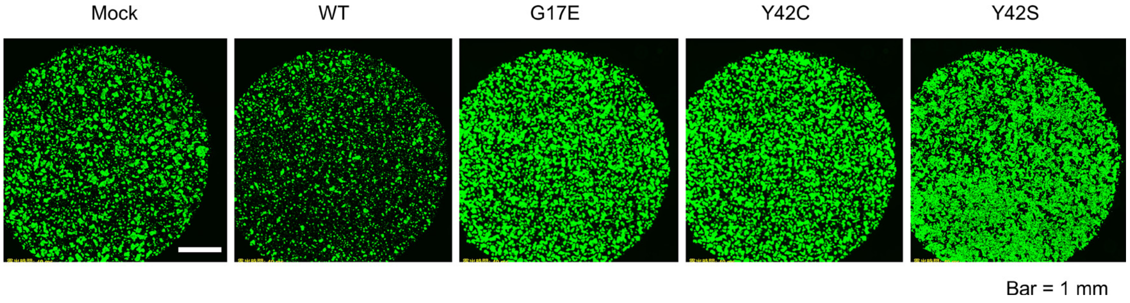

Supplementary Figure 5: Migration activity of MKN74 transfectants with *RHOA* mutations. 48 hrs after cell seeding, fluorescence microscopy was used to detect calcein-incorporated migrating cells. Representative images of each transfectant (n = 3) are shown. Scale bar shows 1 mm.

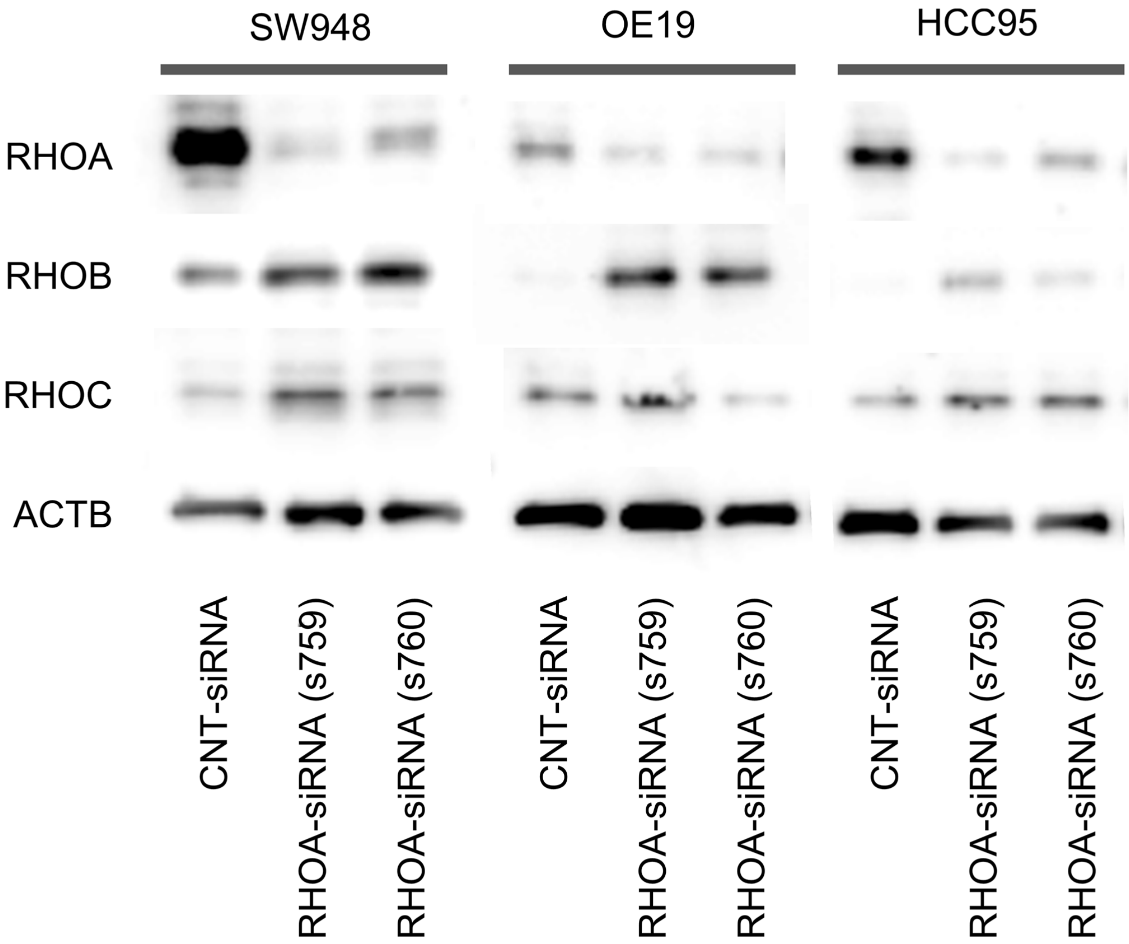

Supplementary Figure 6: Feedback upregulation of RHOB and RHOC proteins after *RHOA* knockdown. Each cell line was seeded in a 6-well plate and then *RHOA*-siRNA was added. Proteins were harvested on day 2 after siRNA treatment. The protein expression levels were detected using western blotting.

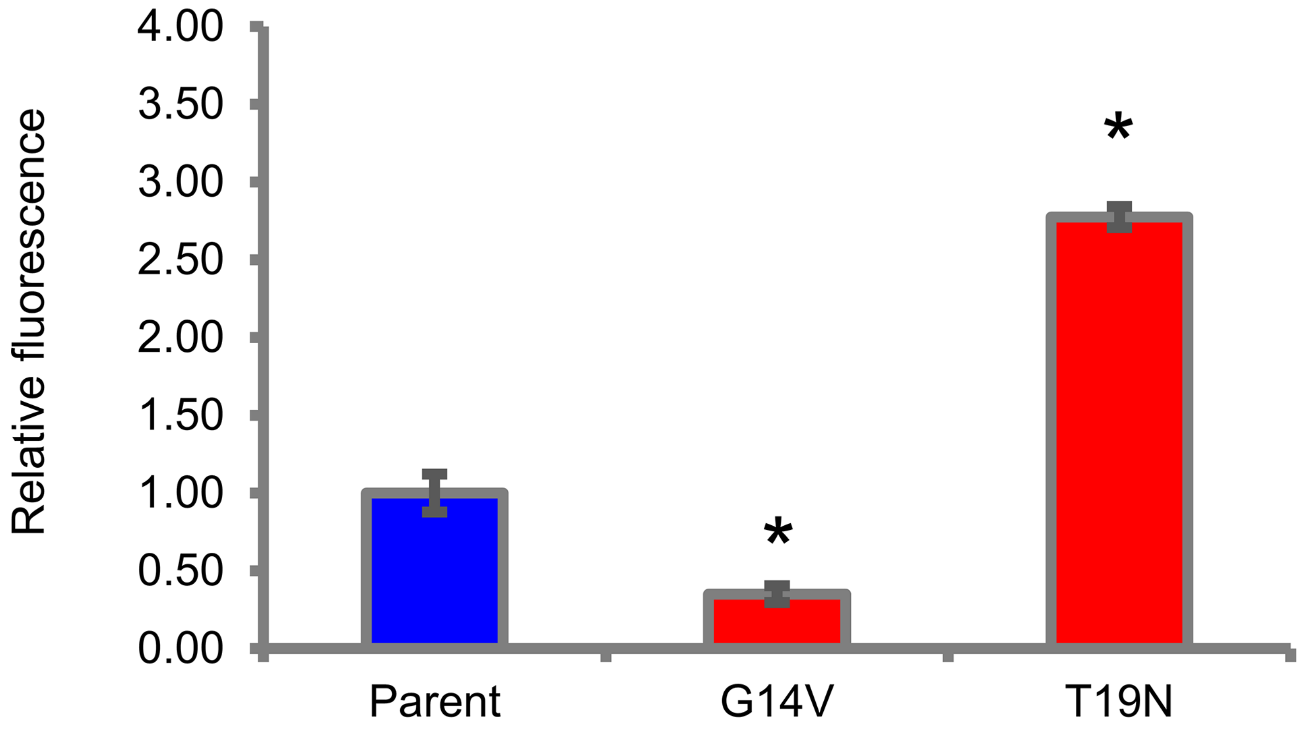

Supplementary Figure 7: Migration activity in MKN74 transfectants of G14V (dominant-active) and T19N (dominant-negative). Cells were seeded in an uncoated chamber and migrated cells after 48 hrs were stained with calcein AM. Data are shown as mean ± SD (n = 3). Statistical significance compared with the parent MKN74 group was determined by Student′s t-test. $^{*}p < 0.05$.

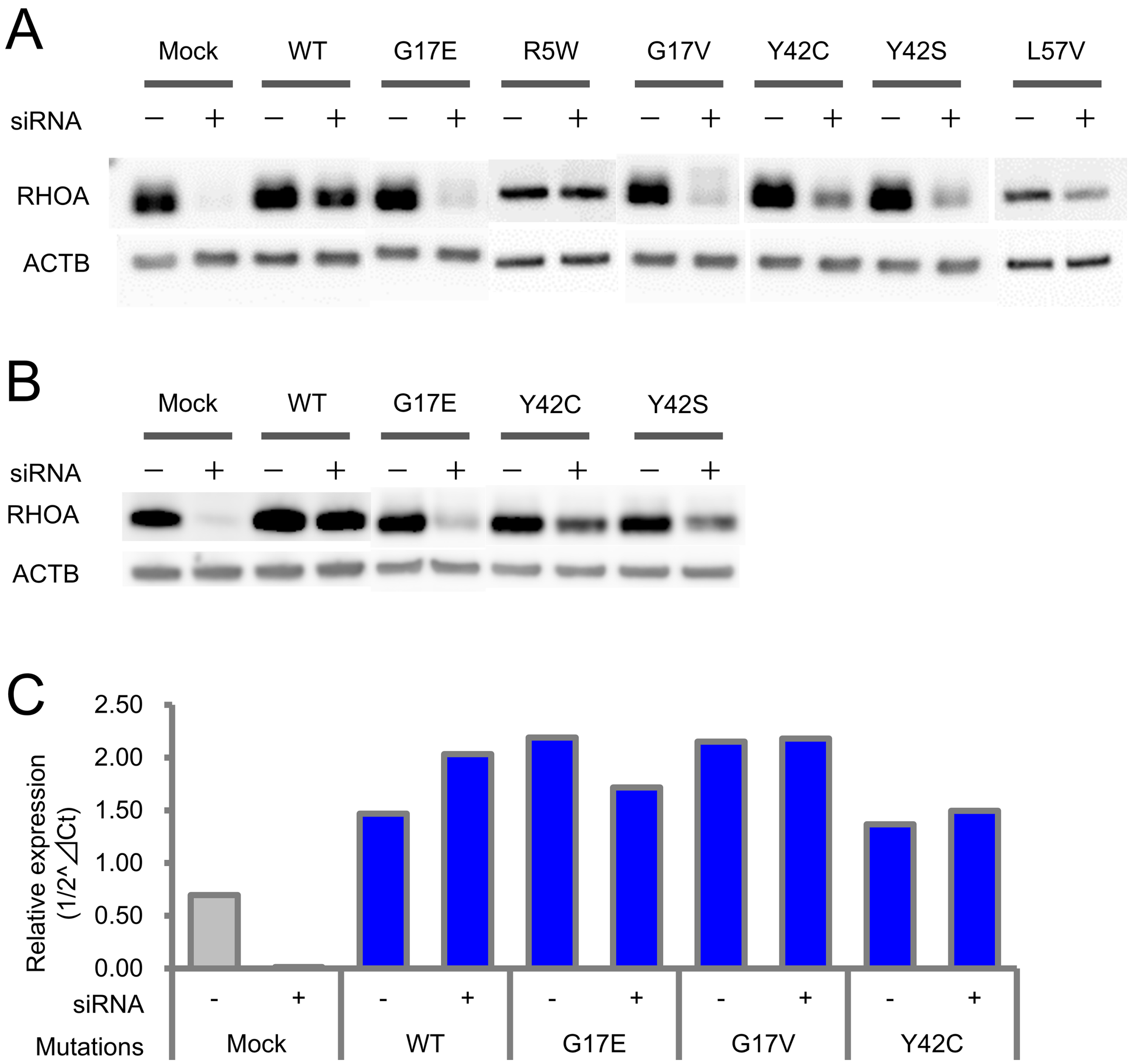

Supplementary Figure 8: Expression analysis of SW948 and MKN74 transfectants. RHOA protein expression of (**A**) SW948 and (**B**) MKN74 transfectants. Cells were seeded in a 6-well plate and then *RHOA*-siRNA was added. Proteins were eluted on day 2, and RHOA protein expression was detected by western blotting. (**C**) *RHOA* mRNA expression of SW948 transfectants of WT, G17E, G17V, and Y42C by qRT-PCR. Cells were seeded in a 6-well plate and then *RHOA*-siRNA was added. After 2 days incubation, mRNA expressions were compared by qRT-PCR. The PCR condition is described in Materials and Methods. Values obtained in qRT-PCR were normalized with *RPS18*.

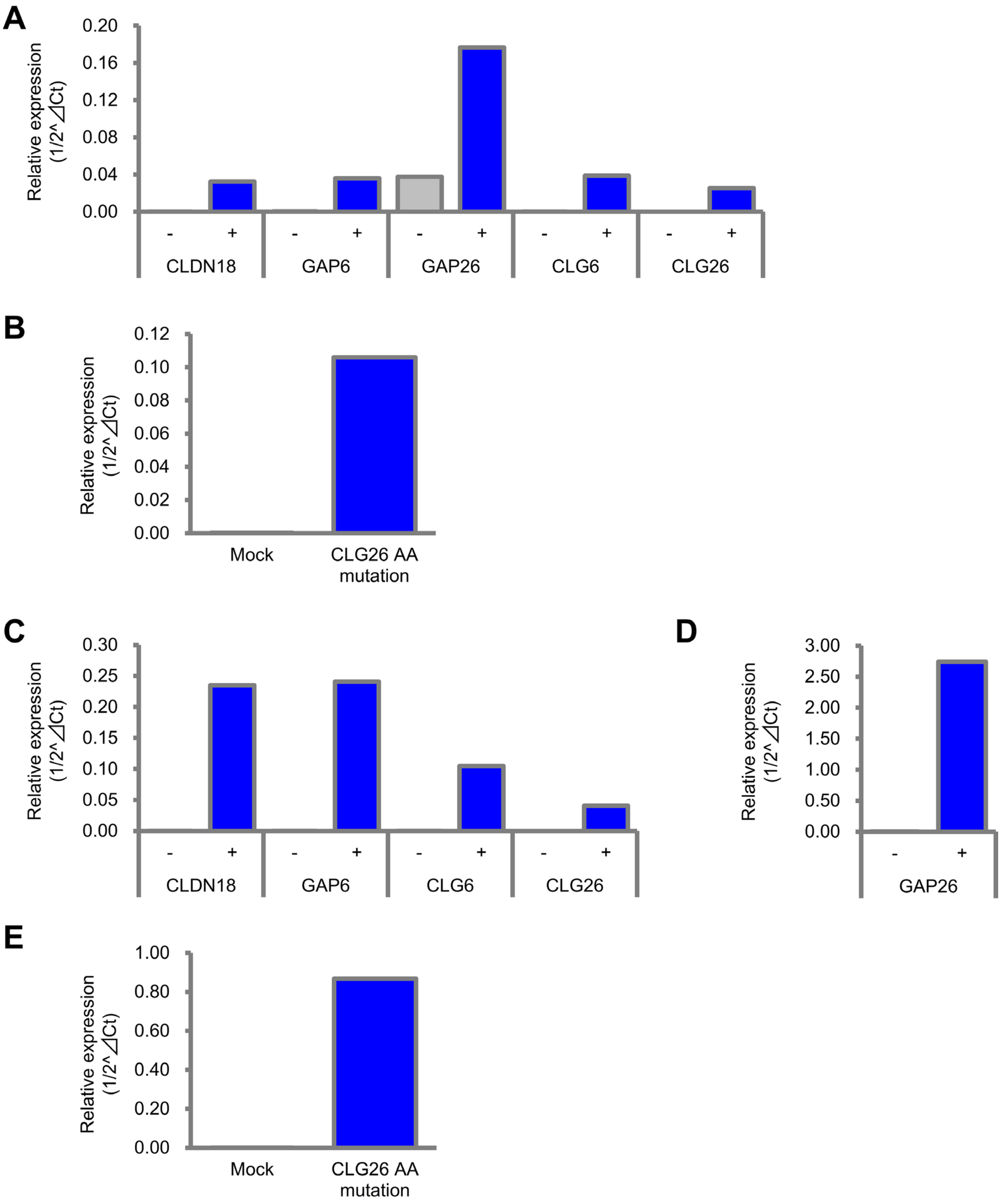

Supplementary Figure 9: mRNA expression of each transfectant of *CLDN18, GAP6, GAP26, CLG6,* and *CLG26* (**A**) SW948, (**C**, **D**) MKN74), and *CLG26* with GAP domain AA mutations (**B**) SW948, (**E**) MKN74). Cells were seeded in a 6-well plate, and after 2 days incubation, mRNAs were eluted and quantitative RT-PCR was performed using each primer. Values obtained in quantitative RT-PCR were normalized with *RPS18*.

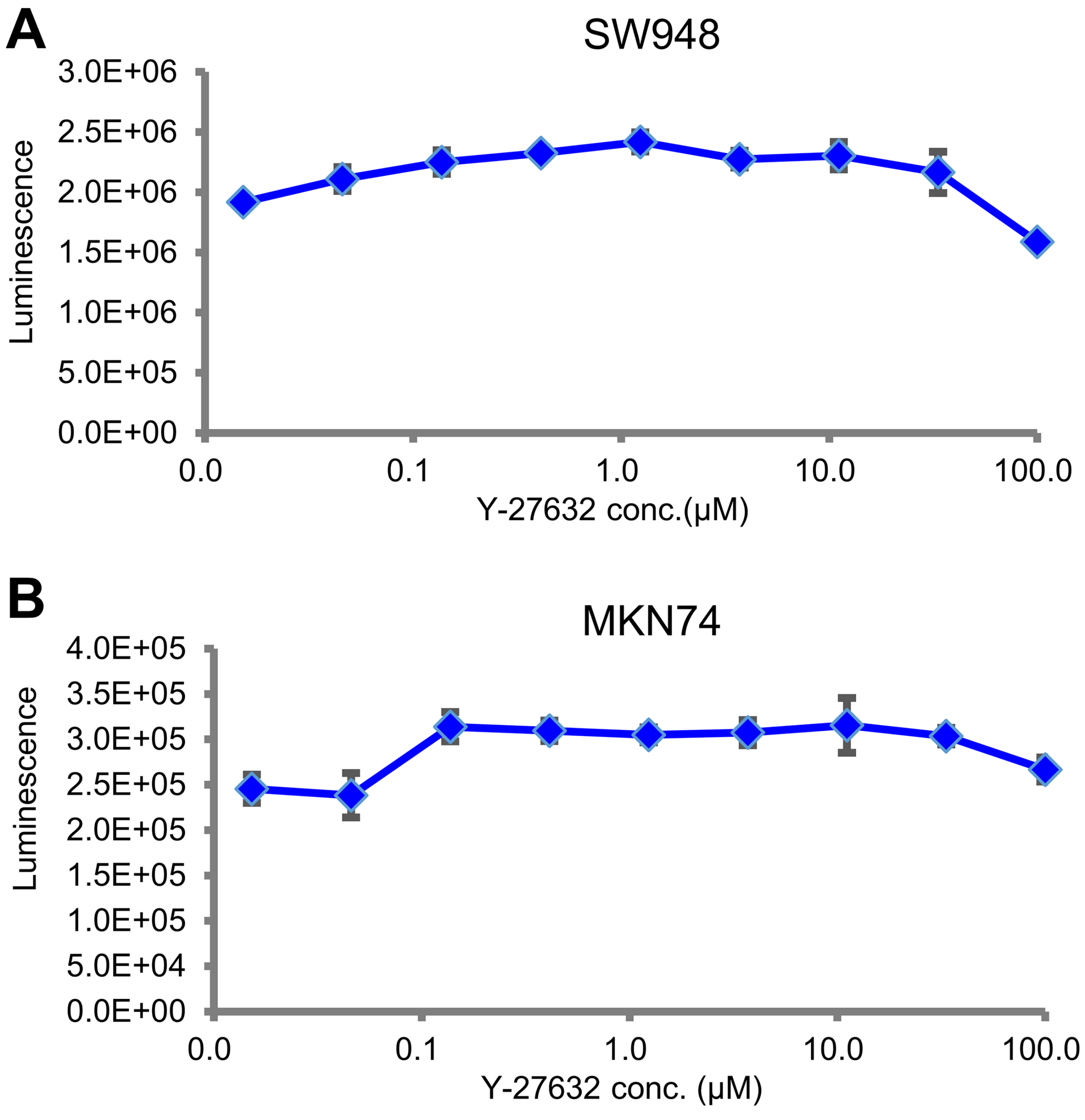

Supplementary Figure 10: Effect of Y-27632 on cell survival. (**A**) SW948 was seeded in a low attachment plate and then treated with various concentrations of Y-27632 for 7 days. (**B**) MKN74 was seeded in a cell culture plate and then treated with various concentrations of Y-27632 for 4 days. The viable cells were measured by a CellTiter-Glo 3D Cell Viability Assay. Data are shown as mean ± SD (*n* = 3).

Supplementary Table 1: Summary of the mutation spectrum of *RHOA* in cancer cell lines

NAME	TISSUE	STATUS	Validated mutations
SK-UT-1	Uterus	Mesodermal tumor	p.R5Q
MDA PCa 2b	Prostate	Adenocarcinoma	p.R5W
KOSC-2	Head & Neck (mouth)	Squamous cell carcinoma	p.R5Q, p.E40Q
CCK-81	Colon	Adenocarcinoma	p.R5Q, p.Y42C
SNU-16	Stomach	Adenocarcinoma	p.R5W, p.F39L
SW948	Colon	Adenocarcinoma	p.G17E
BT-474	Breast	Ductal carcinoma	p.G17E
HCC95	Lung	Squamous cell carcinoma	p.G17A
NUGC-2	Stomach	Adenocarcinoma	p.G17E
NCI-H2170	Lung	Squamous cell carcinoma	p.A3V
GP2d	Colon	Adenocarcinoma	p.Y34C
CJM	Head & Neck (mouth)	Squamous cell carcinoma	p.E40Q
OE19	Esophagus	Adenocarcinoma	p.Y42S
SNU-719	Stomach	Adenocarcinoma	p.A61V
KYSE140	Esophagus	Squamous cell carcinoma	p.Y66D
QG-56	Lung	Squamous cell carcinoma	p.L69P
KNS-62	Lung	Squamous cell carcinoma	p.L69P
MOLT-16	Blood	T acute lymphoblastic leukemia	p.D120N

Mutations were investigated in public databases CCLE and COSMIC and validated by in-house sequencing of hotspot regions.

Supplementary Table 2: DNA and RNA oligonucleotide sequences utilized in this study

(A) siRNA sequences

Target	sense/antisense	siRNA sequences
RHOA (s759)	sense	5′-cuaugauuauuaacgaugutt-3′
	antisense	5′-acaucguuaauaaucauagtt-3′
RHOA (s760)	sense	5′-ggcuuuacuccguaacagatt-3′
	antisense	5′-ucuguuacggaguaaagccct-3′
RHOB (s1575)	sense	5′-ugauaucccuugucuguaatt-3′
	antisense	5′-uuacagacaagggauaucaag-3′
RHOC (s99)	sense	5′-aggacugcauuguuuucuatt-3′
	antisense	5′-uagaaaacaaugcaguccugg-3′
KIF11	sense	5′-ccaucaacacugguaagaatt-3′
	antisense	5′-uucuuaccaguguugaugggt-3′

(B) DNA oligonucleotide sequences for RT-PCR are listed

Target	forward/reverse	Primer sequences
RHOA	forward	5′-gggagctagccaagatgaag-3′
	reverse	5′-gtacccaaaagcgccaatc-3′
RPS18	forward	5′-gtgctgcagccatgtctct-3′
	reverse	5′-gcagtgatggcaaaggctat-3′
CLDN18	forward	5′-gagtgccggggctacttcac-3′
	reverse	5′-ggcaaagatggataccaggagg-3′
GAP6	forward	5′-tcgtcgtcaaagtcaagggaa-3′
	reverse	5′-tgttgaatgccaagttcccca-3′
GAP26	forward	5′-gaactttcgttcacagcaggc-3′
	reverse	5′-gatgaggccagtctttccgt-3′
CLG6	forward	5′-ctggaggcttcaaggccag-3′
	reverse	5′-ggcctgacatgctgttcca-3′
CLG26	forward	5′-cacagaggacgaggtctacaa-3′
	reverse	5′-ctgaagccaatgctgtccaac-3′
